# Whey Protein Films for Sustainable Food Packaging: Effect of Incorporated Ascorbic Acid and Environmental Assessment

**DOI:** 10.3390/polym15020387

**Published:** 2023-01-11

**Authors:** Alaitz Etxabide, Maite Arregi, Sara Cabezudo, Pedro Guerrero, Koro de la Caba

**Affiliations:** 1 BIOMAT Research Group, Escuela de Ingeniería de Gipuzkoa, University of the Basque Country (UPV/EHU), Plaza de Europa 1, 20018 Donostia-San Sebastián, Spain; 2 BCMaterials, Basque Center for Materials, Applications and Nanostructures, UPV/EHU Science Park, 48940 Leioa, Spain

**Keywords:** biowaste valorisation, compostable films, sustainable food packaging, environmental assessment

## Abstract

The management of food waste and by-products has become a challenge for the agri-food sector and an example are whey by-products produced in dairy industries. Seeking other whey valorisation alternatives and applications, whey protein films for food packaging applications were developed in this study. Films containing different amounts (0, 5, 10, and 15 wt%) of ascorbic acid were manufactured via compression-moulding and their physicochemical, thermal, barrier, optical, and mechanical properties were analysed and related to the film structure. Additionally, the environmental assessment of the films was carried out to analyse the impact of film manufacture. Regarding physicochemical properties, both FTIR and water uptake analyses showed the presence of non-covalent interactions, such as hydrogen bonding, between whey protein and ascorbic acid as band shifts at the 1500–1700 cm^−1^ region as well as a water absorption decrease from 380% down to 240% were observed. The addition of ascorbic acid notably improved the UV-Vis light absorbance capacity of whey protein films up to 500 nm, a relevant enhancement for protecting foods susceptible to UV-Vis light-induced lipid oxidation. In relation to the environmental assessment, it was concluded that scaling up film manufacture could lead to a reduction in the environmental impacts, mainly electricity consumption.

## 1. Introduction

The management of food waste and by-products has become a challenge for the agri-food sector (agriculture, farming, and food processing activities), which tackles demanding economic costs for their treatment/disposal, stemming from the increase in food demand/consumption as well as the rigorous environmental regulations. In particular, the dairy industry annually produces millions of tons of by-products, with whey being one of the main components (global production of ~200 million tonnes). Because of its high biological oxygen demand and important organic (lactose) and inorganic (mineral salts, mainly sodium chloride and potassium chloride) nutritional load, whey can cause serious environmental problems (e.g., in aquatic life) if discarded without appropriate treatments [1,2,3]. Whey is mainly composed of water (92%), milk nutrients such as lactose (5%), proteins (1%) (e.g., β-lactoglobulin, α-lactoglobulin, immunoglobulins, serum albumin, and lactoferrin), minerals (1%), fat/lipids (0.1%), and vitamins (B vitamins) [4]. Because whey is a nutrient- and protein-rich compound, around 50% of whey is used as is or in a dry (concentrate or isolate) state to obtain high-added-value compounds for food, pharmaceutical, and chemical industries. However, a high proportion of this by-product is still being wasted/discarded as effluent [1,2,5,6].

Seeking other whey valorisation alternatives and applications, this work is focused on the development of novel biopolymer-based films, aiming at replacing currently used food packaging plastics by greener packaging films, which can be employed to maintain the quality characteristics and extend the shelf life of foodstuffs while having environmental advantages (waste valorisation and biodegradability) over conventional packaging plastics. As a potential approach to improve film properties, such as water resistance and mechanical properties, polyols can be used as plasticisers to prepare flexible films [7,8], with glycerol being the plasticiser used in this work to develop whey-protein-based food packaging. Another way to improve film properties is the inclusion of ascorbic acid (AA), a widely used natural and generally recognised as a safe (GRAS) food ingredient [9,10], which can act as a bioactive compound.

In this study, glycerol-plasticised films were prepared using whey protein isolate (WPI) and their properties were assessed as a function of added AA content. Physicochemical, thermal, barrier, optical, and mechanical properties of the films were analysed and related to the film structure. In addition, the environmental assessment of the films was carried out to evaluate the impact of film manufacturing.

## 2. Materials and Methods

### 2.1. Materials

Whey protein isolate (WPI) was provided by Nutrition Chefs (Donostia-San Sebastian, Spain) and its amino acid analysis was performed with a Biochrom 30+ amino acid analyser physiological system (UK). Glycerol (Gly) was purchased from Panreac (Barcelona, Spain) and ascorbic acid (AA) from Sigma-Aldrich (Madrid, Spain). Milli-Q ultrapure water (Madrid, Spain) was used as the solvent.

### 2.2. Film Preparation

Compression moulding was used to prepare WPI films but a previous step was performed to adjust the pH to 10. The reason for adjusting the pH of the film-forming solution to 10 was to avoid the isoelectric point (pI) of WPI (pI~5). Lower/higher pH values than the pI of whey proteins result in +/− charged proteins, which enable the development of repulsive electrostatic forces. The greater the distance between pH and pI, the stronger the repulsive forces formed; thus, the aggregation of proteins is prevented, which leads to more transparent solutions/films [9,11].

Amounts of 10 g of WPI and 5, 10, or 15 wt% of AA (based on a WPI dry basis) were dissolved in 100 mL of Milli-Q ultrapure water at 80 °C and stirred at 200 rpm for 30 min. Then, Gly was added (50 wt% on a WPI dry basis), the pH was adjusted to 10 (NaOH 1 M), and the solution was heated at 80 °C for other 30 min under magnetic stirring. Afterwards, the solution was freeze-dried for 48 h using an Alpha 1–4 LDplus freeze-drier (INYCOM, Zaragoza, Spain), and the obtained powder was thermally compacted (3 MPa, 2 min) using a Specac hydraulic press, previously heated up to 105 °C. The obtained films were designated as Control, AA5, AA10, and AA15 for those samples prepared with 0, 5, 10, and 15 wt% of AA, respectively. All films were conditioned (25 °C, 50% RH, 48 h) before testing in an ACS Sunrise 700 V bio-chamber.

### 2.3. Film Characterisation

#### 2.3.1. Film Thickness

A Mitutoyo hand-held QuantuMike digimatic (Unceta, Elgoibar, Spain) micrometer was used to measure film thickness to the nearest 0.001 mm (*n* = 3).

#### 2.3.2. Attenuated Total Reflectance Fourier Transform Infrared (ATR-FTIR) Spectroscopy

ATR-FTIR spectra from 4000 to 750 cm^−1^ were performed with a 4 cm^−1^ resolution using a Bruker ALPHA II FTIR spectrometer (Barcelona, Spain).

#### 2.3.3. Water Uptake (WU)

*WU* capacity of the films was calculated by immersing preweighed (*W*_0_) samples (2 cm^2^) in distilled water (50 mL). The samples were weighed again (*W_t_*) at fixed times to obtain the *WU* curves of the films (*n* = 3). *WU* was calculated as follows:

(1) $$WU\left(\%\right)=\frac{\left(W_{t}-W_{0}\right)}{W_{0}}\times 100.\;$$

#### 2.3.4. Thermo-Gravimetric Analysis (TGA) and Differential Scanning Calorimetry (DSC)

TGA (Mettler Toledo TGA/SDTA 851, Madrid, Spain) was performed from 25 to 800 °C and DSC (Mettler Toledo DSC 822) from 5 to 180 °C. Both assays were carried out at 10 °C/min under a N_2_ atmosphere to avoid oxidation reactions.

#### 2.3.5. Colour and Gloss

A Minolta CR-400 Chroma Meter was used to measure (*n* = 10) colour parameters (CIELAB scale): L* = 0 (black) to L* = 100 (white), −a* (greenness) to +a* (redness), and −b* (blueness) to +b* (yellowness) using a white tron (L* = 97.39, a* = 0.03, and b* = 1.77). The total colour difference (ΔE*), referred to as the Control film, was measured as follows:

(2) $$\Delta E^{*}=\sqrt{{\left(\Delta L^{*}\right)}^{2}+{\left(\Delta a^{*}\right)}^{2}+{\left(\Delta b^{*}\right)}^{2}}$$

A Minolta Multi Gloss 268 plus gloss meter was employed to measure gloss (*n* = 10) at a 60° incidence angle, according to ASTM D-523.

#### 2.3.6. Light Absorbance

A V-630 Jasco (Madrid, Spain) ultraviolet-visible (UV-Vis) spectrophotometer was used for the measurement of the light absorbance from 200 to 800 nm.

#### 2.3.7. Water Contact Angle (WCA)

An OCA Dataphysics contact angle meter was used to measure WCA (*n* = 5) by dropping 3 μL of distilled water onto the film surface. Images were captured at 0 and 5 min using SCA20 software.

#### 2.3.8. Water Vapour Permeability (WVP)

A Labthink PERME™ W3/0120 instrument was employed to determine WVP gravimetrically (*n* = 3) at 38 °C and 90% RH, according to ASTM E96-00. Each film was cut in samples of 7.4 cm diameter (test area of 33 cm^2^). First, the water vapour transmission rate (*WVTR*) was calculated as:

(3) $$WVTR\;\left(\frac{g}{s\cdot cm^{2}}\right)=\frac{G}{t\times A}$$

where *G* is the weight change (*g*), *t* is time (h), and *A* is the test area (*cm*^2^). Then, water vapour permeability (*WVP*) was determined as:

(4) $$WVP\left(\frac{g}{cm\cdot s\cdot Pa}\right)=\frac{WVTR\times L}{\Delta P}$$

where *L* is the film thickness (mm) and Δ*P* is the partial pressure difference of water vapour across the film.

#### 2.3.9. Scanning Electron Microscopy (SEM)

A Hitachi S-4800 (Madrid, Spain) scanning electron microscope was used with an acceleration voltage of 15 kV to visualise the film cross-sectional morphology. Samples were placed in a metallic stub and coated with gold under vacuum in an argon atmosphere.

#### 2.3.10. X-ray Diffraction (XRD)

A PANalytic Xpert Pro (Madrid, Spain) equipment with a diffraction unit and Cu-Kα (λ = 1.5418 Å) as a radiation source was used to carry out XRD analysis at 40 kV and 40 mA from 2θ = 2.5 to 50.0°.

#### 2.3.11. Tensile Tests

An Instron 5967 electromechanical testing system (Barcelona, Spain) was used to perform tensile tests. Films were cut with a bone shape of 4.75 mm × 22.25 mm. A tensile load of 500 N and a crosshead rate of 1 mm/min were used to measure mechanical properties (*n* = 10) according to ASTM D638-03.

#### 2.3.12. Environmental Assessment

SimaPro 9.2.0.1 software (Barcelona, Spain) was used to assess the film manufacture environmental impact following the ISO 14040 guidelines and recommendations; the Ecoinvent v3 database was used to obtain the data of energy production, transport, and production of chemicals; the Hierarchist version of ReCiPe 2016, midpoint (H) v1.05, was used to calculate the environmental impacts associated with the film manufacture. First, the functional unit was selected as 10 g of WPI. Then, in the inventory stage, the materials used (WPI, NaOH, glycerol, and water) and the energy (electricity) consumed were considered. As the film manufacture was performed at lab-scale, electricity consumption was estimated by our group. In addition, distilled water production and its transportation to the waste treatment plant after use were considered.

### 2.4. Statistical Analysis

SPSS Statistic 25.0 was used to carry out a one-way analysis of variance (ANOVA) with the level of significance set at *p* < 0.05, determined by post hoc multiple comparisons through Tukey’s test.

## 3. Results and Discussion

### 3.1. Amino Acid Content in WPI

A complete overview of the amino acid (aa) profile as expressed in % is presented in Table 1. The glutamic acid (16.6 %), aspartic acid (11.6%), alanine (9.5%), and leucine (9.1%) contents were the aa with the highest presence in the studied WPI, while arginine (0.9%), histidine/cysteine (1.4%), and methionine (1.7%) were present in the lowest percentages. The WPI used in this study was richer in serine, glycine, proline, and alanine; contained similar percentages of methionine, phenylalanine, histidine, and tyrosine; was poorer in arginine compared with other whey proteins [12,13,14]. It has been seen that differences in amino acid percentages, sequences, and charges may affect the final material properties [15].

### 3.2. Physicochemical Properties of Films

All WPI films were of uniform thickness, as seen in Table 2, as film thickness (~120 µm) did not change significantly (*p* > 0.05) with the increase in AA content from 0 to 15 wt%. The maintenance of film thickness after AA addition indicated a good compatibility between AA and WPI [16].

**Figure 1:**
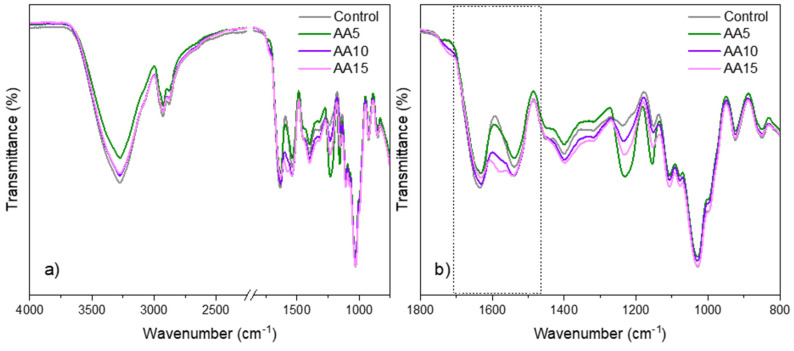
FTIR spectra of WPI films as a function of ascorbic acid (AA) content: (**a**) full spectra from 4000 to 800 cm^−1^ and (**b**) zoom in between 1800 and 800 cm^−1^.

To study the interactions between components that constituted the films, the FTIR spectra are shown in Figure 1. As can be seen in Figure 1a, the spectra displayed signals around 3280 cm^−1^, 2925 cm^−1^, and 2875 cm^−1^, attributed to O-H and stretch vibrations of C-H (CH_2_ and CH_3_), respectively. WPI showed the typical bands of proteins around 1635 cm^−1^, 1540 cm^−1^, and 1230 cm^−1^ assigned to C=O stretching (amide I band), N-H bending (amide II band), and C-N stretching (amide III band), respectively [17,18]. Signals around 940–1150 cm^−1^ were associated with the C-O stretching of carbohydrates [19], present in whey proteins [20]. The main absorption bands of Gly were related to the vibrations of C–C and C–O bonds in the 850–1350 cm^−1^ region [21]. Finally, the bands at 1764, 1675, and 1200–1500 cm^−1^ were associated with C=O stretching, C=C stretching, and C-H deformations of AA, respectively [22].

**Figure 2:**
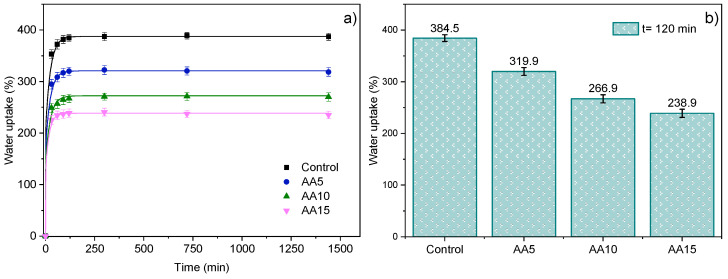
Water uptake capacity of WPI films as a function of ascorbic acid (AA) content (**a**) over time and (**b**) at 120 min (water uptake equilibrium values).

Some band shifts were observed in the 1500–1700 cm^−1^ region (Figure 1b), which were related to non-covalent interactions between WPI and AA. In particular, hydrogen bonding could be formed between carbonyl, hydroxyl, and amino groups of WPI and hydroxyl groups of AA molecules [23]. These outcomes were supported by WU results (Figure 2).

The maximum degree of WU was reached after 120 min of sample immersion (Figure 2b); afterwards, a plateau (equilibrium) was reached (Figure 2a). The WU capacity of Control films was around 380%. These high values could be related to the hydrophilic characteristic of whey proteins. When AA was added, WU values significantly (*p* < 0.05) decreased down to 240% (AA15). This water absorption reduction was related to the physical cross-linking between WPI and AA, as seen by FTIR, as the WU capacity of a sample depends on the amount and nature of intermolecular interactions that, in turn, may reduce its affinity for water [24,25]. It was seen that WU reduction was AA-concentration-dependent, which could be related to the degree of physical interactions promoted between WPI and AA. Considering that a higher degree of cross-linking decreases the water absorbability [26], among the systems assessed in this work (AA5-AA15), this study showed that the maximum degree of intermolecular interactions might be achieved when 15 wt% AA was added into the film formulation (AA15 films).

### 3.3. Thermal Properties

**Figure 3:**
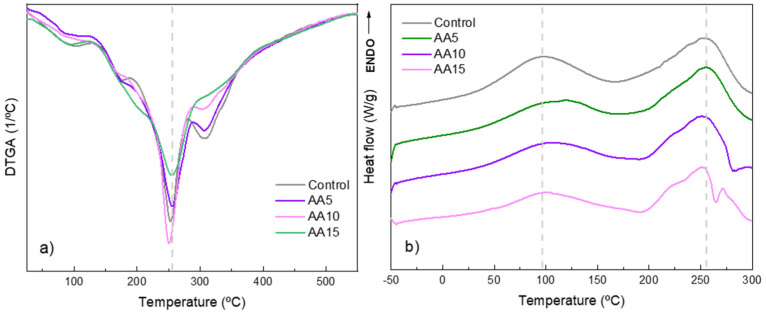
(**a**) TGA derivative (DTGA) and (**b**) DSC thermograms of WPI films as a function of ascorbic acid (AA) content.

The thermal properties of WPI films were determined via TGA and DSC analyses (Figure 3). DTGA curves (Figure 3a) revealed four distinct regions of weight loss changes, irrespective of AA content. First, water evaporation from the films was observed up to 100 °C. Second, the degradation of low-molecular-weight protein components and glycerol occurred between 100 and 200 °C, suggesting interactions (e.g., H-bonds) between WPI and glycerol as this second stage appeared at temperatures higher than the glycerol boiling point (182 °C). Third, the degradation of high-molecular-weight protein fractions took place around 250 °C. In this stage, the DTG peak slightly shifted to lower temperatures as the AA concentration increased. These results could indicate that AA behaves similar to a plasticiser. Finally, the oxidation of partially decomposed proteins and degradation of impurities in whey protein were observed around 320 °C. Degradation temperatures/stages of WPI are in agreement with the literature data obtained for other WPI-based materials [27,28,29,30].

Regarding DSC results, two distinct regions could be observed (Figure 3b): (i) a small endothermic peak at around 100 °C, attributed to water evaporation, and (ii) a major endothermic transition at the region of 225–275 °C. This latter was related to the volatilisation of glycerol as well as to the destruction of ordered molecular structures and thermal decomposition of polypeptide chains. The addition of AA led to slight shifts in the second endothermic peak towards lower temperatures, indicating that there were interactions between WPI and AA that could induce some structural modifications in the protein. AA might act as a plasticiser, leading to these slight changes [11].

### 3.4. Optical Properties

**Figure 4:**
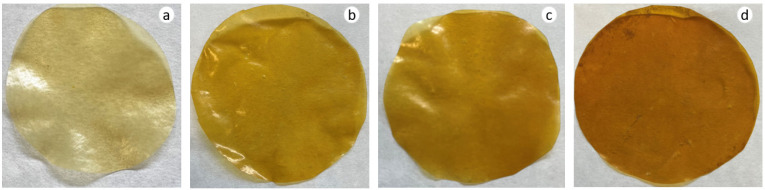
WPI film appearance as a function of ascorbic acid (AA) content: (**a**) 0 (Control), (**b**) 5, (**c**) 10, and (**d**) 15 wt% AA.

The optical properties of the films were assessed based on the colour and gloss parameters (Table 3). The addition of AA induced significant differences in the brightness, redness, and yellowness of films as L* values decreased while a* and b* values increased with AA incorporation. The same trend was observed in AA-containing unripe banana starch films [31]. These colour parameter variations led to notable changes in ΔE*, as compared to Control films, with the difference being higher as the AA concentration increased (Table 3 and Figure 4). Matta and colleagues observed the same outcomes when AA was incorporated in methylcellulose-based films [32]. The present results are also consistent with Agudelo-Cuartas and co-workers [33] who observed that the incorporation of α-tocopherol into whey protein concentrate films increased the ΔE* values of films.

Gloss is directly related to surface roughness: lower gloss values indicate rougher surfaces. Gloss values >70 G.U. (measured at an incidence angle of 60°) indicate glossy and smooth surfaces [34]. In the present study, WPI films presented gloss values of around 23 G.U. and AA addition did not have a significant (*p* > 0.05) effect on the film gloss (Table 3). This means that film surfaces remained rough after AA addition, which could be related to hydrophobic surfaces as rough surfaces present a high surface area, which results in hydrophobic faces [35].

### 3.5. Barrier Properties

**Figure 5:**
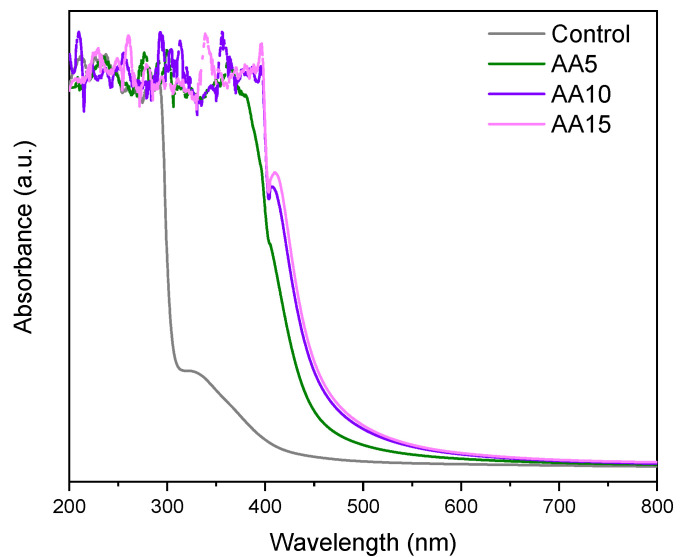
UV-Vis spectra of WPI films as a function of ascorbic acid (AA) content.

The barrier properties of the films were assessed based on light absorbance capacity, water contact angle, and water vapour permeability. The light absorbance capacity of all films is shown in Figure 5. Control films presented high barrier properties between 200 nm and 400 nm (UV region) due to the high content of aromatic amino acids in WPI that can absorb UV light [33]. The addition of AA not only notably improved the UV light absorbance capacity of WPI films but also extended the light protection above 500 nm (Vis region). A similar trend was observed in starch films with the incorporation of AA [31], and it was suggested that AA addition conferred opacity, which provided a better barrier against the light. In our study, changes in optical properties were also observed (Figure 4 and Table 3), resulting in films with better UV-Vis light protection. Considering that the emitted UV-Vis light may induce food oxidation, especially in high-lipid foodstuffs, the films prepared in this study, particularly AA10 and AA15 films, could have potential for food packaging to delay lipid oxidation caused by UV-Vis light [28].

WCA is a good indicator of the film tendency to absorb water; thus, this measurement can be used to assess the water barrier properties of the films. Considering that initial WCA values may not precisely represent the wettability nature of a surface, as they may be time-dependent [36], measurements were taken at 0 and 5 min after the drop was placed on the film surface, with the aim of better evaluating the wetting characteristics of the samples. The film surface wettability data (Table 4) showed that AA addition significantly (*p* < 0.05) increased the surface wettability of WPI films when compared with the Control film, but no significant (*p* > 0.05) differences were found among all AA-containing films (AA5-AA15). As shown in the literature [37], AA also affected the wettability of potato starch films as WCA values decreased with AA addition. Despite the WCA value decrease, all films remained hydrophobic (WCA > 90°) at 0 min, which could be related to film roughness, while they became hydrophilic after 5 min (WCA < 90°), regardless of AA concentration. The increase in surface wettability of films with AA addition could be caused by the hydrophilic nature of the active component itself, as well as by the exposure of some hydrophilic groups toward the film surface. A further decrease in WCA values over time (up to 5 min) could be related to the swelling/water absorption of film, as seen in other works on WPI-based edible films [38].

Control films presented WVP values of around 4.6 × 10^−12^ g·cm^−1^·s^−1^·Pa^−1^ and the addition of AA resulted in negligible WVP changes (*p* > 0.05). Although WVP values increased up to 7.12 × 10^−12^ g·cm^−1^·s^−1^·Pa^−1^ with 15 wt% AA content (Table 4), they were close and in the same order of magnitude. This insignificant increase could be related to the fact that AA contains a large amount of OH groups that promoted the water vapour diffusion through the film [17]. However, WVP values did not change significantly, as also seen in the literature on whey protein concentrate/pullulan composite films containing a bacteriophage [39].

### 3.6. Film Structure

**Figure 6:**
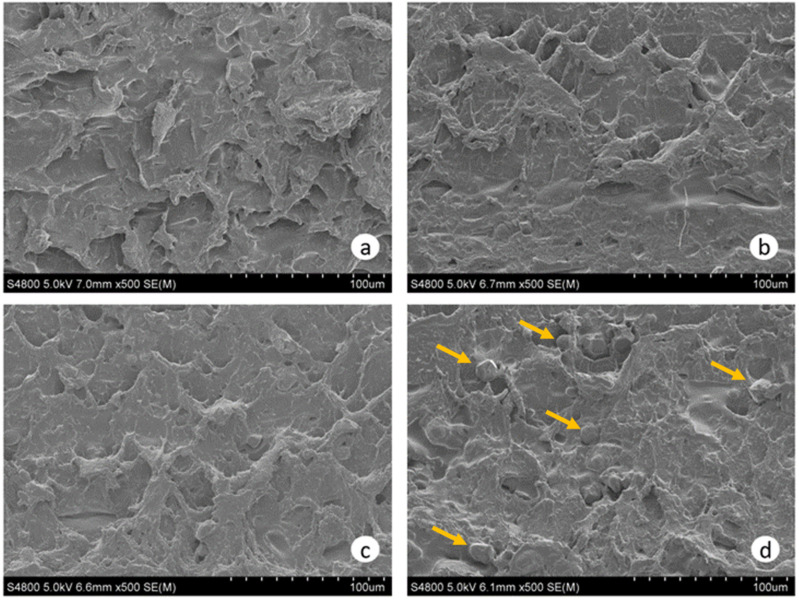
SEM images of WPI films as a function of ascorbic acid (AA) content: (**a**) 0 (Control), (**b**) 5, (**c**) 10, and (**d**) 15 wt% AA. Yellow arrows point out small granules observed in WPI films.

Film structure was assessed using SEM and XRD techniques. SEM images of the film cross-section can be seen in Figure 6. In general, a continuous and homogeneous microstructure without any pores was observed in films without AA (Control) and with 5 wt% AA (AA5), as can be seen in Figure 6a,b. This view slightly changed after the inclusion of 10 wt% AA as few small granules could be observed in AA10 films (Figure 6c). As the AA content increased (AA15), the presence of these granules increased (arrows in Figure 6d). Similar results were observed in ferulic-acid-containing soy-protein-isolate-based films, where aggregations were formed when high amounts of ferulic acid were added [40]. Structure changes in gelatin films from rabbit skin were also observed when rosemary acid was incorporated [41]. The observed structure modifications were related to protein–polyphenol interactions and protein self-aggregation induced by the excessive ferulic acid/rosemary acid addition into protein-based films. The incorporation of increased AA concentrations in this work affected the morphology of WPI films in the same manner.

**Figure 7:**
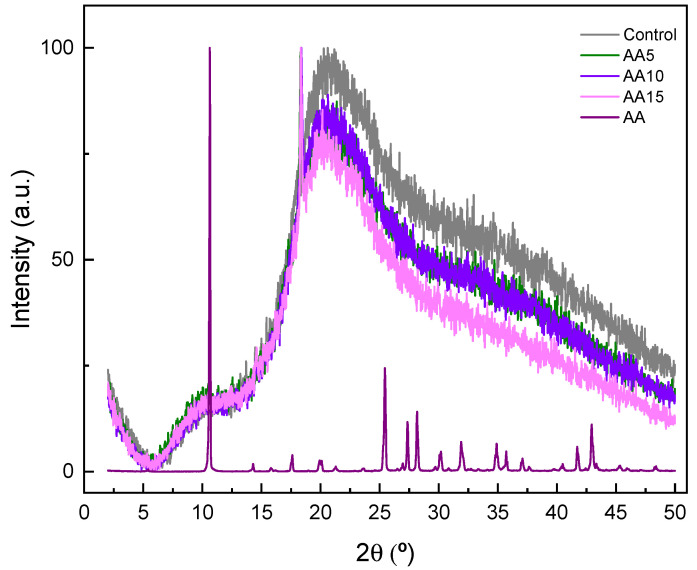
XRD patterns for ascorbic acid (AA) and WPI films as a function of AA content.

In XRD diffractograms, sharp peaks are related to the crystalline fractions of the analysed material, while a broad and diffuse background represents the amorphous parts of the studied sample [42]. Considering that the films assessed in the present work exhibited a typical amorphous diffraction pattern (Figure 7): a small and broad peak around 2θ = 10° and a more intensive less-broaden peak around 2θ = 20°, similar patterns were observed in other WPI films, such as for WPI and xylan composite films [30]. Regarding AA addition, XRD patterns did not change when AA was present in films, regardless of its content. Some studies have shown that interactions between WPI and vitamins (AA is vitamin C) may not affect the tertiary structure of WPI [23].

### 3.7. Mechanical Properties

The addition of AA increased EB significantly (*p* < 0.05), but decreased significantly (*p* < 0.05) EM and TS values, as shown in Table 5. This was because AA is a small molecule, consisting of multiple hydroxyl groups, which could behave as a plasticiser, disrupting H-bonds between neighbouring protein chains [43]. Higher AA contents led to further changes due to interactions between polymer networks being further reduced. A similar trend was observed in WPI/cassava starch films where TS decreased and EB increased due to the plasticising effect of starch [17]. Similarly, whey-protein-based films incorporated with natamycin and α-tocopherol were less resistant/stiff and more stretchable films with the addition of the active compounds [33]. Further, the addition of low-molecular-weight galactooligosaccharide and xylooligosaccharide had a similar effect on WPI films, related to the development of an intermolecular spacing effect within WPI chains, similar to that observed when plasticisers are used [16].

### 3.8. Environmental Assessment

**Figure 8:**
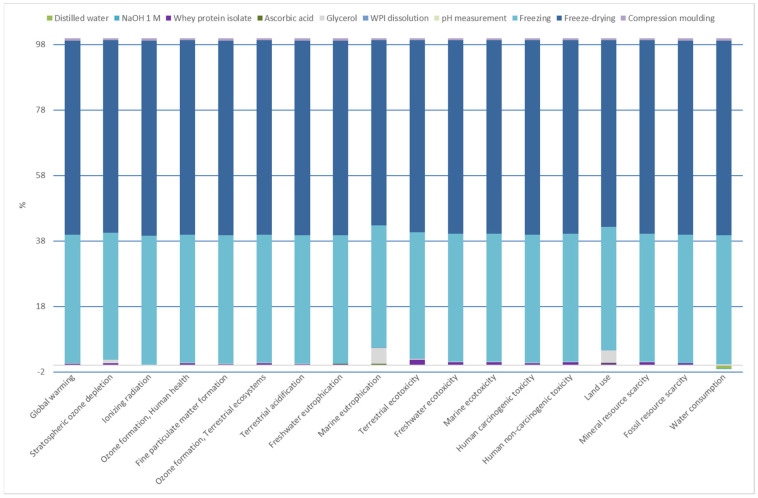
Relative contributions of each impact category for WPI film manufacture.

The main goal of the environmental assessment carried out in this study was to evaluate the environmental impact of WPI film manufacture to identify the environmental load related to each process of turning raw materials into films. In this context, the energy consumed in the manufacturing stages and the materials used to prepare films were considered as inputs to determine the environmental burden related to each process in the production of WPI films at lab-scale. As shown in Figure 8, freezing and freeze-drying processes were the main contributors (95%) to the impact load. The energy used in these processes, specifically electricity consumption, had a critical role in the environmental impact, regardless of the impact category. The environmental assessment identified these two processes as the most relevant indicators. In addition to them, the use of glycerol represented around 5% of the environmental impact in both land use and marine eutrophication categories. This was because glycerol is a co-product in the esterification process of soybean oil production to obtain biodiesel. Ecoinvent considers an allocation factor of 92% to soybean oil and 8% to glycerol. Thus, when glycerol was added into the film-forming formulation, the impacts of soybean cultivation (e.g., use of diesel, machines, fertilisers, and pesticides) were considered, giving rise to the environmental impact on land use and marine eutrophication categories.

The environmental assessment of WPI films indicated that the abovementioned processes should be improved and optimised to reduce the environmental load associated with the WPI film manufacture. As these films were developed at a laboratory scale, scaling up processes could lead to achieving the goal of the reduction in the environmental impacts abovementioned. As comparison of lab-scale products with industrial production does not provide realistic data, due to the fact that optimised processes at industrial scale, as well as scaling effects, lead to better resource efficiency [44], a comparative environmental assessment with a conventional food packaging was not carried out. However, the environmental assessment of an early research state product is helpful in providing comparative information related to the different processes involved in the product preparation at lab-scale, which is the aim of the environmental assessment carried out in this work.

## 4. Conclusions

The WPI used in this study contained a high presence of glutamic acid (16.6%), aspartic acid (11.6%), alanine (9.5%), and leucine (9.1%) amino acids. Regarding WPI-based films prepared via compression-moulding, non-covalent interactions such as H-bonds between WPI and AA were observed through FTIR analysis (band shifts at 1500–1700 cm^−1^ region), and water absorption was also decreased from 380% down to 240%. The addition of AA notably improved the UV-Vis light absorbance of WPI films up to 500 nm. This enhancement in light barrier properties would have significant potential in food packaging, particularly in foods susceptible to UV-Vis light-induced lipid oxidation. AA addition improved the elongation at break due to the role as plasticiser of AA and did not compromise the gloss, water vapour permeability, structure, and thermal properties of the films. These properties make WPI a promising candidate for the replacement of fossil commodity polymers used for food packaging applications. Based on the environmental assessment of WPI films, scaling up film manufacture could lead to the reduction in the environmental load associated with the electricity consumption.

## Figures and Tables

**Table 1:** The amino acid content (%) in WPI.

Amino Acids	%
Aspartic acid	11.578
Threonine	6.715
Serine	7.165
Glutamic acid	16.588
Proline	7.497
Glycine	3.412
Alanine	9.514
Cysteine	1.435
Valine	5.618
Methionine	1.705
Isoleucine	4.754
Leucine	9.075
Tyrosine	2.461
Phenylalanine	2.636
Histidine	1.390
Lysine	7.536
Arginine	0.923

**Table 2:** Thickness of WPI films as a function of AA content.

Films	Thickness (µm)
Control	119.86 ± 3.47 ^a^
AA5	120.75 ± 6.68 ^a^
AA10	122.62 ± 3.96 ^a^
AA15	123.52 ± 3.52 ^a^

^a^ Two means followed by the same letter in the same column are not significantly (*p* > 0.05) different through Tukey’s multiple range test.

**Table 3:** Colour parameters (L*, a*, b*, and ΔE*) and gloss values of WPI films as a function of ascorbic acid (AA) content.

Films	L*	a*	b*	ΔE*	Gloss (G.U.)
Control	91.27 ± 1.00 ^a^	−4.24 ± 0.19 ^a^	35.97 ± 2.92 ^a^		22.95 ± 6.43 ^a^
AA5	78.35 ± 1.02 ^b^	10.01 ± 1.08 ^b^	53.72 ± 0.83 ^b^	26.17 ± 1.64 ^a^	25.11 ± 2.76 ^a^
AA10	74.24 ± 1.16 ^c^	15.24 ± 1.53 ^c^	62.63 ± 1.26 ^c^	37.15 ± 2.21 ^b^	22.16 ± 1.32 ^a^
AA15	76.05 ± 1.02 ^d^	13.49 ± 1.02 ^d^	64.07 ± 0.69 ^c^	36.56 ± 1.26 ^b^	22.73 ± 1.79 ^a^

^a–d^ Two means followed by the same letter in the same column are not significantly (*p* > 0.05) different through Tukey’s multiple range test.

**Table 4:** Water contact angle (WCA) and water vapour permeability (WVP) values of WPI films as a function of ascorbic acid (AA) content.

Films	WCA (°) t = 0 min	WCA (°) t = 5 min	WVP·10^12^ (g·cm^−1^·s^−1^·Pa^−1^)
Control	105.6 ± 4.9 ^a^	88.2 ± 4.1 ^a^	4.57 ± 1.26 ^a^
AA5	91.7 ± 4.2 ^b^	71.7 ± 3.5 ^b^	5.98 ± 1.29 ^a^
AA10	88.8 ± 4.2 ^b^	69.4 ± 3.7 ^b^	5.89 ± 1.58 ^a^
AA15	88.4 ± 3.2 ^b^	68.8 ± 2.8 ^b^	7.12 ± 0.94 ^a^

^a–b^ Two means followed by the same letter in the same column are not significantly (*p* > 0.05) different through Tukey’s multiple range test.

**Table 5:** Elastic modulus (EM), tensile strength (TS), and elongation at break (EB) of WPI films as a function of AA content.

Films	EM (MPa)	TS (MPa)	EB (%)
Control	24.71 ± 1.65 ^a^	1.49 ± 0.08 ^a^	18.83 ± 1.25 ^a^
AA5	20.82 ± 1.07 ^b^	1.43 ± 0.07 ^a^	23.54 ± 0.75 ^b^
AA10	19.59 ± 0. 92 ^b^	1.12 ± 0.08 ^b^	23.14 ± 0.92 ^b^
AA15	16.98 ± 1.23 ^c^	1.09 ± 0.06 ^b^	23.00 ± 0.81 ^b^

^a–c^ Two means followed by the same letter in the same column are not significantly (*p* > 0.05) different through Tukey’s multiple range test.

## Data Availability

The data presented in this study are available on request from the corresponding author.
